# The impact of trade frictions on the financial vulnerability of Chinese households

**DOI:** 10.1371/journal.pone.0333713

**Published:** 2026-04-10

**Authors:** Xiaoling Xue, Yunfei Hao, Jiankang Feng, Linlin Han

**Affiliations:** 1 School of Business, University of Jinan, Jinan, China; 2 Journal Publishing Office of Humanities and Social Sciences, Shandong University, Jinan, China; 3 Institute of State Governance, Shandong University, Jinan, China; 4 School of Economics, Shandong Normal University, Jinan, China; 5 School of Economics and Management, Qilu Normal University, Jinan, China; Southern Illinois University Carbondale, UNITED STATES OF AMERICA

## Abstract

In recent years, escalating trade frictions have exerted substantial shocks on the financial security of Chinese households. Drawing on data from the China Family Panel Studies (CFPS) for 2014–2022, this study constructs a standard difference-in-differences (DID) model, taking the 2018–2019 U.S. tariff hikes as a quasi-natural experiment. We systematically assess the impact of trade frictions on household financial vulnerability and explore the underlying mechanisms. The findings indicate: (1) trade frictions significantly increase the financial vulnerability of Chinese households; (2) households in urban and export-oriented regions, households with less-educated heads, and those lacking business income, property income, or social security coverage are disproportionately affected; and (3) Trade frictions weaken household financial resilience mainly through employment and income shocks, with price and savings channels playing secondary roles, while asset volatility and especially debt burdens exert the weakest effects. This study provides critical evidence for understanding how external macroeconomic shocks are transmitted to the household level. The findings also offer practical insights for enhancing household economic resilience, strengthening risk-coping capacity, and supporting the broader strategy of expanding domestic demand.

## 1. Introductionand literature review

Understanding how external trade shocks influence household financial vulnerability has become increasingly important amid rising global economic uncertainty. Although existing research has examined the macroeconomic and firm-level impacts of the U.S.–China trade conflict, far less is known about how such shocks are transmitted to the household sector and how they affect families’ financial resilience. Addressing this gap is crucial for understanding how trade disruptions shape household economic stability.

Drawing on nationally representative micro panel data from the China Family Panel Studies (CFPS) for 2014–2022, this study provides micro-level evidence on how trade frictions affect the financial vulnerability of Chinese households, identifies the mechanisms through which these effects are transmitted, and documents the heterogeneity across different types of households. Methodologically, we implement a difference-in-differences (DID) strategy that exploits the 2018–2019 tariff hikes as a quasi-natural experiment, allowing us to causally estimate the impact of trade shocks on household financial outcomes.

In this study, household financial vulnerability refers to a condition in which households exhibit insufficient liquidity buffers or shock-absorption capacity to cope with unexpected income losses or expenditure surges, thereby facing an elevated risk of financial distress.

Using the 2018–2019 tariff hikes as a quasi-natural experiment to identify the causal effects of trade shocks on household financial outcomes. Moreover, mechanism analyses show that employment and income disruptions constitute the primary transmission channel, whereas price, savings, asset, and especially debt channels play comparatively weaker roles. These findings contribute new empirical evidence to the growing literature on household-level responses to external economic disturbances. In addition, we find that trade frictions significantly increase the financial vulnerability of Chinese households. In addition, substantial heterogeneity across household groups: families living in urban and export-oriented regions, households headed by individuals with low education levels, and those lacking income diversification or social protection face significantly greater financial vulnerability under trade shocks.

Existing literature has primarily focused on the macroeconomic and firm-level consequences of trade frictions. Scholars have examined their effects on economic growth [1], international trade [2], supply chain resilience [3], industries [4], regional development [5], capital markets [6–7], and household consumption [8]. Other studies have explored innovation [9], firm cash holdings [10], digital technology adoption [11], and firm-level export adjustments [12]. However, the household-level dimension remains comparatively underexplored, and these studies largely focus on aggregate or firm outcomes rather than on how trade shocks directly affect families.

More recent work has begun to consider household responses to trade shocks. For example, Zhang et al. (2025) [13]employ a two-country HANK model to investigate the distributional effects of trade frictions, while Cai et al. (2024) [14] investigate how tariff uncertainty shapes household portfolio choices. On the consumption side, Farrokhi et al. (2022) [15] and Cícero & Heras-Recuero (2026) [16] map trade-induced changes in prices and inequality into household expenditure patterns and luxury-goods imports, respectively. Although the above studies shed light on how trade frictions influence specific dimensions of household financial behavior—such as consumption decisions or portfolio adjustments—they typically examine only a single behavioral margin and thus fall short of providing a comprehensive assessment of overall household financial vulnerability. In particular, they do not simultaneously account for multiple elements such as income risk, liquidity buffers, debt burdens, and exposure to expenditure shocks. Moreover, they lack causal identification based on longitudinal microdata that could reveal how major trade frictions alter the probability that households fall into a financially vulnerable state. In contrast, this paper treats household financial vulnerability as an integrated outcome and leverages Chinese household panel data to causally identify the impact of trade frictions on the likelihood of entering financial vulnerability.

Findings from the household finance literature further underscore the importance of examining financial vulnerability. Existing studies indicate that financial vulnerability is closely associated with income volatility, debt burdens, liquidity constraints, and fluctuations in asset prices. Under external shocks, households are more likely to fall into a trap of insufficient savings, rising debt burdens, and reliance on high-risk borrowing [17]. In the Chinese context, household wealth is highly concentrated in housing assets, and asset price volatility directly affects consumption and financial stability through balance sheet and wealth effects [18]. It can thus be inferred that trade frictions, as a representative form of external shock, are likely to exacerbate household financial vulnerability through three major channels: employment and income, inflation, and asset price fluctuations. Yet, the absence of causal identification and heterogeneity analysis based on micro-level household data leaves a crucial gap in understanding how macro shocks are transmitted to and amplified within the household sector.

As the U.S.–China trade conflict escalated—marked by multiple rounds of tariff increases and expanded export-control measures—the resulting shocks significantly affected labor demand, wage levels, living costs, and asset markets. Given these transmission channels, household financial stability is likely to respond more directly and more sensitively than macroeconomic or firm-level indicators suggest. Understanding household-level responses thus provides an important complement to broader analyses of trade disruptions.

The rest of the paper is organized as follows. Section 2 presents the theoretical framework and research hypotheses. Section 3 describes the research design, including data, variables, and empirical strategy. Section 4 reports the main empirical results. Section 5 conducts mechanism tests, while Section 6 provides heterogeneity analyses. Section 7 concludes with policy implications.

## 2. Theoretical framework and research hypotheses

Between 2018 and 2019, the United States imposed four rounds of high tariffs on Chinese exports, covering goods worth US$370 billion. These tariffs dealt substantial shocks to labor-intensive sectors, such as manufacturing, mining, information transmission, computer services, and software industries [1]. By undermining China’s export competitiveness, the tariffs triggered a sharp drop in export volumes and pushed many export-oriented firms to reduce or suspend production [2]. As profits contracted, firms typically responded by reducing working hours, imposing compulsory leave, cutting jobs, lowering wages, reducing bonuses and benefits, and shrinking demand for informal employment (e.g., gig workers and temporary employees). These measures heightened unemployment risks—especially for informal workers—and substantially reduced wage income.

Wage income represents the core and most stable source of household cash flow in China, surpassing the stability of business or property income [19]. Stable cash flow helps households cover fixed costs, preserve creditworthiness, and avoid costly borrowing, which enhances financial resilience [20]. Consequently, households employed in industries directly exposed to trade shocks face elevated risks of unemployment and wage reduction, which amplify their financial vulnerability.

**Hypothesis 1:** Trade frictions exert significant adverse effects on household employment and income, thereby increasing household financial vulnerability.

The imposition of tariffs also directly reduced the price competitiveness of Chinese exports, leading to declining export volumes and forcing some firms to redirect exports to the domestic market [2]. Yet, mismatches between product specifications or designs and domestic demand required additional investment in production line modifications and the establishment of new domestic distribution networks, thereby raising the prices of comparable goods in domestic markets. At the same time, China’s retaliatory tariffs increased the import costs of U.S. goods, while Chinese firms dependent on imported intermediate goods or raw materials faced rising procurement costs [1]. To preserve profitability, domestic producers passed on these costs to consumers, further driving up retail prices. As a result, households encountered higher everyday living expenses. Cavallo et al. (2021) provide international evidence on tariff-induced price increases, a pattern consistent with what Chinese households faced during the trade conflict [5].

Trade frictions thus simultaneously raised prices and unemployment risks, compelling households to shoulder higher rigid expenditures amid declining incomes. To sustain essential spending on housing, healthcare, and education, household savings rates dropped sharply, and precautionary savings buffers eroded [8]. Many households resorted to debt financing to cover shortfalls, which led to surging household debt levels and rising debt-to-income ratios. Although Dynan & Kohn (2007) examine U.S. households, their findings capture general mechanisms—such as rising leverage in response to income shocks—that are also applicable to households facing trade-induced financial stress in China [21]. Furthermore, reduced income and unemployment constrained access to formal credit, pushing households toward high-cost informal borrowing and worsening debt structures. Similarly, Banerjee & Duflo (2019), while not focused on China, provides widely recognized evidence on how credit constraints intensify financial fragility, a mechanism that can also arise among Chinese households under trade shocks [22]. The depletion of savings, combined with high leverage and deteriorating debt composition, ultimately undermined households’ ability to cope with shocks such as illness or job loss, thereby intensifying their financial vulnerability.

**Hypothesis 2a:** Trade frictions increase household consumption expenditures while reducing household savings, thereby raising household financial vulnerability.

**Hypothesis 2b:** Trade frictions heighten household debt burdens and worsen debt structures, thereby raising household financial vulnerability.

Finally, U.S.–China trade frictions have aggravated asset price volatility through two channels. First, U.S. tariffs on Chinese exports reduced the profit margins of export-oriented firms, weakened investor expectations, and amplified stock market volatility [6–7]. Consequently, the value of household financial assets fluctuated with market swings. Second, export-dependent cities experienced industrial chain relocation shocks, leading to declines in housing prices. As housing constitutes the largest share of household wealth in China [13], falling property prices directly eroded household wealth. Asset price volatility reduced households’ ability to liquidate assets for emergency needs, further exacerbating financial vulnerability.

**Hypothesis 3**: Trade frictions amplify asset price volatility, thereby increasing household financial vulnerability.

## 3. Research design

### 3.1 Model specification

To assess the impact of trade frictions on household financial vulnerability, we construct the following difference-in-differences (DID) model:


$${\text{LEV}}_{\text{it}}=\alpha +\beta Treated_{i}*Post_{t}+\gamma X_{it}+\lambda t+\mu i+\varepsilon_{it}$$ (1)

where *LEV*_*it*_ denotes the financial vulnerability of household *i* in year *t*; *Treated*_*i*_ is a treatment indicator; *Post*_*t*_ is a post-event indicator; *X*_*it*_ denotes control variables at the individual, household, and regional levels; and *λ*_*t*_ and *μ*_*i*_ represent year and household fixed effects, respectively.

Since the dependent variable *LEV*_*it*_ is binary (taking values 0 or 1), this study employs a Linear Probability Model (LPM) within a two-way fixed-effects Difference-in-Differences (DID) framework for the baseline estimation. The model is estimated using ordinary least squares (OLS), and the coefficient β can be directly interpreted as the percentage-point change in the probability that a household becomes financially vulnerable as a result of trade frictions.

### 3.2 Data and variable definitions

The study uses data from the 2014, 2016, 2020, and 2022 waves of the CFPS. After excluding households with heads under 16 years old, extreme outliers, and cases with missing key variables, the final sample consists of 21,337 households.

- Dependent variable: Following Brunetti et al. (2016) [23], financial vulnerability is defined using a financial buffer margin, calculated as household income minus essential living costs and debt service. Households with a negative buffer are coded as financially vulnerable (1), and otherwise as not vulnerable (0).
- Independent variable: The treatment group comprises households whose heads worked in industries directly targeted by U.S. tariffs (manufacturing, mining, IT services, and agriculture). The event period is defined as 2020 and 2022, while 2014 and 2016 serve as pre-treatment periods.
- Control variables: These include demographic characteristics (household head’s age, gender, marital status, education), household structure (child and elderly ratios, household size, income), and regional characteristics (urban/rural status, province).

Descriptive statistics reveal that 37.5% of households are financially vulnerable, while 30.22% of household heads are employed in tariff-exposed industries. On average, households consist of three members, the head is 41 years old, 79.28% are married, and 24.68% hold college or higher degrees (shown in *Appendix 1* S1 File).

## 4. Empirical results and analysis

### 4.1 Baseline regression

This section estimates Equation (1) using a two-way fixed-effects Linear Probability Model (LPM), to assess the effects of trade frictions on household financial vulnerability. The results are presented in Table 1. Columns (1)–(4) report regressions that progressively incorporate household demographic characteristics, household economic variables, and regional-level controls in addition to the core explanatory variables. Across all specifications, the estimated coefficients of the interaction term are significantly positive, indicating that trade frictions increase the likelihood of household financial vulnerability. Specifically, trade frictions increased the probability of households falling into financial vulnerability by 4%. These findings confirm that trade frictions exacerbate the financial fragility of Chinese households.

**Table 1:** Baseline regression.

	*LEV*	*LEV*	*LEV*	*LEV*
	(1)	(2)	(3)	(4)
Treated*Post	0.0411^**^	0.0405^**^	0.0370^**^	0.0373^**^
	(0.0186)	(0.0186)	(0.0183)	(0.0183)
*age*		-0.0023^***^	-0.0030^***^	-0.0031^***^
		(0.0006)	(0.0006)	(0.0006)
*sex*		-0.0099	-0.0008	-0.0001
		(0.0101)	(0.0099)	(0.0099)
*edu*		0.0081	0.0286^*^	0.0303^*^
		(0.0167)	(0.0166)	(0.0166)
*Uy*		0.0944	0.0378	0.0378
		(0.0640)	(0.0638)	(0.0638)
*Uo*		0.013	-0.0092	-0.0127
		(0.0248)	(0.0243)	(0.0243)
*familysize*		-0.006	0.0061	0.0043
		(0.0048)	(0.0048)	(0.0048)
*marital*		0.0868^***^	0.1123^***^	0.1074^***^
		(0.0170)	(0.0169)	(0.0169)
*urban*				0.0002
				(0.0025)
*lny*			-0.1034^***^	-0.1023^***^
			(0.0066)	(0.0066)
*Treated*	-0.0362^**^	-0.0374^**^	-0.0336^**^	-0.0322^**^
	(0.0147)	(0.0147)	(0.0144)	(0.0144)
*Post*	-0.0573^***^	-0.0518^***^	0.0061	0.0078
	(0.0097)	(0.0111)	(0.0115)	(0.0116)
*Province fixed effects*				Yes
*Household fixed effects*	Yes	Yes	Yes	Yes
*Time fixed effects*	Yes	Yes	Yes	Yes
*Coefficient*	0.4036^***^	0.4439^***^	1.5401^***^	1.4607^***^
	(0.0055)	(0.0279)	(0.0759)	(0.0929)
*N*	21,337	21,337	21,337	21,337
*R* ^*2*^	0.004	0.0077	0.0370	0.0393

*Notes: (1) All regressions use robust standard errors. (2) ***, *, and * *denote significance at the 1%, 5%, and 10% levels, respectively. Standard errors are reported in parentheses. The same notation applies to subsequent tables.*

In addition to the core treatment effect, several control variables exhibit statistically significant associations with household financial vulnerability. Age is negatively associated with vulnerability across specifications, implying that older household heads are less likely to experience financial fragility, possibly due to more stable income flows or accumulated assets. Marital status is positively associated with vulnerability, indicating that married households may encounter higher financial stress due to greater consumption commitments or childcare responsibilities. Furthermore, household income (lny) shows a strong negative relationship with financial vulnerability, consistent with the expectation that higher-income households possess stronger buffers and a greater capacity to absorb economic shocks. Together, these results highlight the heterogeneous financial risks faced by different household groups and underscore the importance of demographic and socioeconomic characteristics in shaping household financial resilience.

### 4.2 Parallel trends assessment

The validity of the difference-in-differences (DID) method hinges on the assumption that the treatment and control groups exhibit parallel pre-treatment trends. To verify this assumption, we estimate the following event-study model:


$${\text{LEV}}_{\text{it}}=\alpha +\beta_{\text{1}}D_{\text{i}2014}+\beta_{\text{2}}D_{\text{i}2016}+\beta_{\text{3}}D_{\text{i}2020}+\beta_{\text{4}}D_{\text{i}2022}+\gamma X_{it}+\lambda t+\mu i+\varepsilon_{it}$$ (2)

where interaction terms capture pre- and post-event effects (e.g., *Treated × Pre2014*, *Treated × Pre2016*, *Treated × Pre2020*, *Treated × Pre2022*). Control variables mirror those in the baseline regressions, including demographic, household, and regional characteristics, with year and household fixed effects also included.

The null hypothesis (H0) of the parallel trends test is that the pre-treatment coefficients are jointly insignificant. The regression results yield a p-value of 0.2264, failing to reject the null. This indicates that treatment and control groups exhibited no significant trend differences prior to the imposition of U.S. tariffs, thereby validating the parallel trends assumption.

### 4.3 Placebo tests

#### (1) Temporal placebo test.

To ensure that the estimated effects are not driven by spurious time-related factors, we introduce a fictitious treatment period by assuming that the Sino–U.S. trade frictions occurred in 2016. Re-estimating Equation (1) under this assumption (Table 2, Column 1) shows that the interaction term is statistically insignificant. This result supports the credibility of the actual treatment effect.

**Table 2:** Placebo tests.

	*LEV*	*LEV*
	(1)	(2)
Treated*Post	0.0203	-0.0318
	(0.0203)	(0.0226)
*Control variables*	Yes	Yes
*Province fixed effects*	Yes	Yes
*Household fixed effects*	Yes	Yes
*Time fixed effects*	Yes	Yes
*Coefficient*	1.4395^***^	1.4487^***^
	(0.0891)	(0.0929)
*N*	21,337	21,337
*R* ^*2*^	0.0392	0.0390

#### (2) Industry placebo test.

To further rule out the possibility that “industry-specific shocks unrelated to the tariffs” (i.e., unrelated shocks) may confound the identification, we select four industries——s education, healthcare and social security, culture and entertainment, and public administration—as placebo treatment groups. These sectors were completely unaffected by tariff policies during the U.S.–China trade conflict, and their industry dynamics are entirely unrelated to the tariff shock. The results (Table 2, Column 2) again show insignificant interaction terms. This suggests that the baseline estimates are not driven by sector-specific trends or omitted variables. Therefore, the placebo test based on unrelated industries provides additional support that the observed increase in household financial vulnerability is indeed attributable to the genuine trade shock.

### 4.4 Sensitivity analysis

To mitigate potential misclassification arising from defining treatment status solely based on the household head’s industry, we conduct two additional sensitivity checks. The first check redefines the treatment group by classifying a household as treated if **any** working member is employed in a tariff-exposed industry.Table 3 (Column 1) shows that, under this alternative definition, the coefficient on the interaction term (Treated*Post) remains positive, and its direction is fully consistent with the baseline estimate. Although the coefficient becomes statistically insignificant, this attenuation is expected because incorporating all working members inevitably introduces greater measurement error and weakens the effective intensity of trade-shock exposure. More importantly, the sign and economic interpretation remain unchanged, indicating that the baseline finding—that trade frictions increase the probability of households falling into financial vulnerability—is not driven by relying solely on the industry of the household head.

**Table 3:** Sensitivity analysis.

	*LEV*	*LEV*
	(1)	(2)
Treated*Post	0.0146	0.0829^**^
	(0.0169)	(0.0419)
*Control variables*	Yes	Yes
*Province fixed effects*	Yes	Yes
*Household fixed effects*	Yes	Yes
*Time fixed effects*	Yes	Yes
*Coefficient*	1.4564^***^	1.5594^***^
	(0.0928)	(0.1802)
*N*	21,337	6,344
*R* ^*2*^	0.0389	0.0322

The second sensitivity check restricts the sample to single-income-source households, for which the household head’s industry more accurately reflects the family’s dominant income source, thereby substantially reducing the risk of treatment misclassification in multi-earner households. Column (2) shows that, within this more stringent sample, the interaction term remains positive and becomes statistically significant at the 5% level, closely aligning with the baseline conclusion.

Taken together, these two sensitivity checks validate the robustness of the baseline results from different perspectives. Regardless of whether the treatment definition is broadened (any member exposed) or the sample is narrowed (single-income-source households), the estimated effects remain consistent in sign and economic meaning. This confirms that alternative definitions of household exposure do not overturn the core finding that trade frictions significantly heighten household financial vulnerability, thereby reinforcing the robustness of the main conclusion.

### 4.4 Robustness checks

#### (1) Alternative treatment group definitions.

Two adjustments are made to the treatment group to test robustness. First, given that U.S. tariffs disproportionately targeted manufacturing products, we restrict the treatment group to households with heads employed in manufacturing. Second, recognizing that households in high-export dependency provinces (e.g., the Yangtze River Delta and Pearl River Delta) are more exposed to tariff shocks, we redefine the treatment group as households located in these regions whose heads are employed in tariff-exposed industries (manufacturing, IT services, mining, and agriculture). The results, reported in Table 4 (Columns 1–2), consistently yield significantly positive coefficients for the core interaction term, reinforcing the conclusion that trade frictions exacerbate household financial vulnerability.

**Table 4:** Robustness test results.

	Adjusted treatment group (Sample 1)	Adjusted treatment group (Sample 2)	Alternative dependent variable	Expanded sample
	(1)	(2)	(3)	(4)
Treated*Post	0.0436^**^	0.1000^***^	0.0403^**^	0.0424^**^
	(0.0195)	(0.0285)	(0.0163)	(0.0184)
*Control variables*	Yes	Yes	Yes	Yes
*Province fixed effects*	Yes	Yes	Yes	Yes
*Household fixed effects*	Yes	Yes	Yes	Yes
*Time fixed effects*	Yes	Yes	Yes	Yes
*Coefficient*	1.4647^***^	1.4591^***^	0.2831^***^	1.4655^***^
	(0.0929)	(0.0929)	(0.0050)	(0.0779)
*N*	21,337	21,337	21,337	21,676
*R* ^*2*^	0.0396	0.0402	0.0047	0.0315

#### (2) Alternative dependent variable.

Following Zang and Liu (2025) [24], we redefine household financial vulnerability as a condition where financial deficits are negative and liquid assets are insufficient to cover them. Here, the financial deficit is computed as total household income minus essential consumption expenditures and debt obligations, while liquid assets comprise household cash and deposits. The coverage threshold is set at three months, following Xu et al. (2022) [25]. The regression results using this alternative measure (Table 4, Column 3) remain significantly positive, confirming the robustness of the baseline findings.

#### (3) Expanded sample.

Since U.S. tariffs were imposed in multiple rounds between 2018 and 2019, restricting post-treatment observations to 2020 and 2022 may overlook short-term effects. Moreover, the COVID-19 pandemic after 2020 may confound results. To capture the immediate impact of tariff implementation while isolating it from pandemic effects, we include 2018 as part of the post-treatment period. The results (Table 4, Column 4) remain robust, with trade frictions continuing to exhibit a significant positive effect on household financial vulnerability.

#### (4) Re-estimation using logit and probit models.

Given that the dependent variable *LEV*_*it*_ is binary (0/1), we further re-estimate Equation (1) using Logit and Probit specifications as a robustness check.

The general form of the nonlinear model is specified as:


$${\text{Pr(LEV}}_{\text{it}}=1|⬝)=F(\alpha +\beta Treated_{i}*Post_{t}+\gamma X_{it}+\lambda t+\mu i+\varepsilon_{it})$$ (3)

where F(•) denotes the logistic cumulative distribution function for the Logit model and the standard normal cumulative distribution function for the Probit model.

For the Logit specification, we apply the conditional fixed-effects Logit estimator (Chamberlain, 1980) [26] to eliminate individual-specific effects and avoid the incidental parameter bias. For the Probit model, we employ the correlated random-effects (CRE) approach following Mundlak (1978) [27], which controls for the potential correlation between individual effects and covariates.

The results (Table 5) show that, regardless of whether the FE-Logit or CRE-Probit model is employed, the estimated coefficient of the interaction term (*Treated × Post*) remains consistent in both sign and statistical significance with the baseline regression. This indicates that the positive impact of trade frictions on household financial vulnerability is robust across different model specifications.

**Table 5:** Robustness test results: re-estimation using logit and probit models.

	FE-Logit	CRE-Probit
	(1)	(2)
Treated*Post	0.1013^**^	0.1013^**^
	(0.0473)	(0.0473)
*Control variables*	Yes	Yes
*Province fixed effects*	Yes	Yes
*Household fixed effects*	Yes	Yes
*Time fixed effects*	Yes	Yes
*Coefficient*	3.7930^***^	3.7930^***^
	(0.2285)	(0.2285)
*N*	20,792	20,792
*Treated = 0*	0.3910	0.3890
*Treated = 1*	0.3410	0.3443
*Post = 0*	0.3678	0.3691
*Post = 1*	0.3886	0.3856

Additionally compute and report the average marginal effects (AMEs) in percentage-point terms. The AME estimates indicate that exposure to trade frictions raises the probability of household financial vulnerability by approximately 5.0 percentage points in the FE-Logit model and 4.5 percentage points in the CRE-Probit specification. These percentage-point marginal effects closely align with the baseline LPM estimates and confirm that the positive effect of trade frictions on financial vulnerability is robust across alternative nonlinear estimators.

## 5. Mechanism tests

### 5.1 Income channel: Employment and wage shocks

When facing demand contractions, firms typically adjust working hours before resorting to layoffs. We therefore use changes in weekly working time (Ltime) to capture the employment impact of Sino–U.S. trade frictions on Chinese households. As reported in Column (1) of Table 6, Trade frictions are significantly associated with a reduction in household weekly working hours, indicating a negative effect on household employment. A decline in working hours is commonly viewed as a leading indicator of reduced income, implying a weakening of households’ risk-buffering capacity and, consequently, a potential rise in financial vulnerability. Trade frictions also depress export volumes of tariffed products. To contain costs, affected firms lay off workers or cut wages, exposing employees to heightened unemployment risk and income uncertainty. Workers who become unemployed—or experience steep wage cuts—often turn to low-return flexible jobs (e.g., ride-hailing, street vending) to stabilize income, which alters household income composition. We focus on the shares of wage income in total income (yw) and business income in total income (yo) to characterize this adjustment. Columns (2) – (3) of Table 6 show that Trade frictions are significantly associated with a decrease in the share of wage income and an increase in the share of business income. Because wage income is less volatile and more stable than business income, this shift implies lower overall income stability and higher income risk, thereby potentially increasing the likelihood that households fall into financial vulnerability, consistent with the expectations of Hypothesis 1.

**Table 6:** Mechanism tests.

	*Ltime*	*y*_*w*_	*y*_*o*_	*lnc*	*srate*	*w_chg*	*dy*
	(1)	(2)	(3)	(4)	(5)	(6)	(7)
Treated* Post	-2.5182^**^	-0.0211^**^	0.0241^***^	0.0497^**^	-0.0925^***^	0.1964^**^	19.3501^*^
	(1.1608)	(0.0105)	(0.0069)	(0.0248)	(0.0342)	(0.0886)	(11.6823)
*Control variables*	Yes	Yes	Yes	Yes	Yes	Yes	Yes
*Province fixed effects*	Yes	Yes	Yes	Yes	Yes	Yes	Yes
*Household fixed effects*	Yes	Yes	Yes	Yes	Yes	Yes	Yes
*Time fixed effects*	Yes	Yes	Yes	Yes	Yes	Yes	Yes
*Coefficient*	11.3155^***^	1.1328^***^	0.0649^**^	8.8411^***^	-10.8892^***^	-3.5611^***^	-9.3774
	(0.3681)	(0.0706)	(0.0283)	(0.1930)	(0.3208)	(0.5374)	(27.7607)
*N*	20,481	21,325	21,249	20,362	20,352	12,589	19,966
*R* ^*2*^	0.1919	0.0568	0.0308	0.1667	0.3126	0.0414	0.0009

### 5.2 Price channel: Higher daily living expenditures

To test whether trade frictions raise household expenditures through higher prices, we examine their impact on household consumption (lnc). Column (4) of Table 6 shows that Trade frictions are significantly associated with an increase in households’ daily consumption expenditures. This suggests that household living costs may have increased in the context of the external shock. The rise in consumption expenditures can compress disposable savings and may intensify financial pressure when income does not grow correspondingly, thereby potentially increasing household financial vulnerability. This result is consistent with the logic of Hypothesis 2a.

### 5.3 Asset channel: Reduced wealth accumulation and greater asset-value volatility

Household wealth buffers are a first line of defense against shocks. The buffer-stock theory posits that adequate wealth reserves can offset income–expenditure gaps, reduce reliance on high-interest borrowing, and delay fire sales of assets, thereby preventing an escalation of vulnerability [28]. To examine changes in wealth accumulation, we use the household saving rate (sratio). Column (5) of Table 6 indicates that trade frictions are significantly associated with a decline in household saving rates, implying that households’ wealth buffers may be weakened under the shock, which is consistent with the direction proposed in Hypothesis 2a.

Combining the collateral channel with buffer-stock considerations [16] suggests that stable asset values prevent credit tightening (via collateral shrinkage) and reduce the risk of distress sales—both crucial for household liquidity. We proxy asset-value stability by asset volatility (w_chg), with lower values indicating greater stability. Household asset volatility is measured as the relative change in household assets between the current survey wave and the preceding one. Column (6) of Table 6 shows that Trade frictions are significantly associated with higher asset volatility, suggesting that household asset values may become more unstable under the shock. When asset values fluctuate more widely, households’ liquidity buffers may be eroded, thereby increasing their financial vulnerability. This result is consistent with the mechanism proposed in Hypothesis 3.

### 5.4 Credit-constraint channel: Heavier household debt burdens

We measure household debt burden by the debt-to-income ratio (dy) and assess how trade frictions affect repayment pressure. Column (7) of Table 6 shows that trade frictions are associated with a higher *dy*, suggesting that households may face increased repayment pressure following the shock. This finding is consistent with the mechanism proposed in Hypothesis 2b, whereby rising debt burdens may weaken household liquidity buffers and thereby heighten financial vulnerability.

Overall, Trade frictions increase household financial vulnerability through multiple channels, but the strength of these mechanisms differs. The employment and income channels are the most robust, with significant declines in working hours and deteriorations in income composition. The price and savings channels also respond significantly, as higher living costs and reduced savings further weaken households’ financial buffers. By contrast, asset volatility and debt burdens, while directionally consistent with expectations, exhibit weaker significance and effect sizes and thus function as secondary mechanisms.

## 6. Heterogeneity analysis

### 6.1 Regional heterogeneity

#### (1) Urban–rural differences.

Given China’s dual urban–rural structure, households differ markedly in asset composition, income sources, social-security coverage and generosity, and consumption patterns; heterogeneous effects are therefore expected. We estimate separate regressions for urban and rural samples (Table 7, Columns 1–2). Trade frictions significantly increase financial vulnerability among urban households, whereas the effect for rural households is statistically insignificant.

**Table 7:** Regional heterogeneity results.

	Urban	Rural	Domestic-demand-led provinces	High-export-dependency provinces
	(1)	(2)	(3)	(4)
Treated*Post	0.0639^***^	-0.0166	0.0156	0.0941^***^
	(0.0229)	(0.0360)	(0.0219)	(0.0359)
*Control variables*	Yes	Yes	Yes	Yes
*Household fixed effects*	Yes	Yes	Yes	Yes
*Time fixed effects*	Yes	Yes	Yes	Yes
*Coefficient*	1.3015^***^	1.8774^***^	1.6077^***^	1.3039^***^
	(0.1127)	(0.3051)	(0.1072)	(0.1786)
*N*	14,043	7,294	16,134	5,203
*R* ^*2*^	0.0429	0.0514	0.047	0.0306

Although rural households typically have lower incomes and weaker social protection—and migrant workers form the backbone of manufacturing—several features explain the empirical pattern. First, tariff shocks primarily hit export-oriented manufacturing (machinery and electronics, textiles and apparel, furniture), which is concentrated in urban and coastal industrial zones. Employment in these sectors is dominated by urban-registered workers and a subset of migrants counted among urban residents; thus, urban households face more direct employment and income shocks. By contrast, many migrants occupy non-core, temporary, or agency roles. While exposed, they retain rural land as an economic safety net and can return home to buffer shocks. Second, rural households often draw on multiple sources—farming, local casual work, migrant earnings, and policy transfers—making them less sensitive to a single wage shock than urban households, which are more wage-dependent. Third, heightened uncertainty increases capital-market volatility and slows (or reverses) house-price growth in some cities. Because real estate is the core asset for urban households and mortgages are common, declines or stagnation in housing values weaken asset buffers; rigid mortgage payments then amplify fragility. Rural housing is typically self-built and mortgage-free, limiting exposure through this channel.

#### (2) Openness of regional economies.

Structural differences across regions imply heterogeneous sensitivity to external shocks. We split households into those in high–export-dependency provinces (e.g., Yangtze River Delta, Pearl River Delta) and those in demand-led inland provinces, and re-estimate Equation (1) for each group (Table 7, Columns 3–4). Under trade shocks, financial vulnerability rises significantly among households in high-openness regions whose heads work in tariff-exposed industries, whereas the effect is smaller and insignificant in demand-led inland provinces. The former rely more heavily on export-oriented sectors, transmitting external shocks more directly to household finances; the latter depend mainly on domestic markets, dampening the pass-through of trade frictions.

### 6.2 Household-characteristic heterogeneity

#### (1) Demographic heterogeneity: Education level of household head.

Prior research suggests that education significantly affects occupational choice, skill adaptability, and income resilience. Consequently, households with different education levels may vary in sensitivity to trade shocks. We divide the sample into households with heads holding a college degree or above (high education) and those with heads below college education (low education) and re-estimate Equation (1) for each group (Table 8).

**Table 8:** Heterogeneity by household head’s education.

	High-education households	Low-education households
Treated*Post	0.0218	0.0445^**^
	(0.0475)	(0.0216)
*Control variables*	Yes	Yes
*Household fixed effects*	Yes	Yes
*Time fixed effects*	Yes	Yes
*Province fixed effects*	Yes	Yes
*Coefficient*	0.9686^***^	1.6251^***^
	(0.2000)	(0.1309)
*N*	5,267	16,070
*R* ^*2*^	0.0329	0.0536

Results indicate that, among low-education households, the coefficient of the core interaction term is 0.0445 and significant at the 5% level, whereas it is insignificant among high-education households. This suggests that trade frictions disproportionately affect low-education households, significantly increasing their financial vulnerability, while the impact on high-education households is not statistically significant.

Several mechanisms explain this divergence. Low-education households are concentrated in highly substitutable and demand-elastic jobs, leaving them more exposed to unemployment and income risks under trade shocks. Human capital constraints make reemployment difficult. Their asset portfolios also tend to be undiversified due to limited financial literacy and risk aversion, concentrated in illiquid assets such as housing, which cannot easily be liquidated in response to shocks. By contrast, high-education households are more likely to work in countercyclical sectors such as R&D and finance, where jobs are less substitutable and wages more stable. They also exhibit stronger reemployment capacity due to transferable skills, facilitating horizontal mobility. Moreover, high-education households generally demonstrate greater financial literacy and risk tolerance, adopting diversified asset portfolios that enhance liquidity reserves and generate property income. These advantages strengthen their ability to withstand trade shocks, reducing the likelihood of falling into financial vulnerability.

#### (2) Economic heterogeneity: Household income structure.

Households differ substantially in income sources, asset-allocation capacity, and exposure channels, which may condition the effects of trade shocks. Because income composition is central to household economic characteristics, we focus on differences in income sources. In China, households typically derive income from wages, business activities, property, transfers, and other sources. Among these, wages, business, and property income dominate: nearly 90% of households earn wages, 32.43% report business income, and only 19.51% report property income (Appendix 2 in S1 File).

To test for heterogeneous effects, we group households by whether they report business income and property income, respectively, and re-estimate Equation (1) (Table 9). Columns (1)–(2) show that trade frictions significantly increase financial vulnerability among households without business income, while effects on those with business income are insignificant. Columns (3)–(4) show a similar pattern for property income: the effect is significant for households without property income but not for those with property income. These findings highlight that diversified income sources can substantially mitigate the adverse financial effects of trade shocks, with business income providing a slightly stronger buffer than property income.

**Table 9:** Heterogeneity by household income structure.

	Households without operating income	Households with operating income	Households without property income	Households with property income
	(1)	(2)	(3)	(4)
Treated*Post	0.0470^**^	-0.0191	0.0448^**^	0.017
	(0.0231)	(0.0479)	(0.0221)	(0.0523)
*Control Variables*	Yes	Yes	Yes	Yes
Time Effects	Yes	Yes	Yes	Yes
Individual Effects	Yes	Yes	Yes	Yes
*Coefficient*	1.3184^***^	2.0890^***^	1.4717^***^	1.4128^***^
	(0.1163)	(0.2101)	(0.1129)	(0.2937)
*N*	14,417	6,844	17,174	4,114
*R* ^*2*^	0.0337	0.0681	0.043	0.0452

### 6.3 Heterogeneity in social protection coverage: Health insurance

Social protection serves as a critical buffer against external shocks by providing unemployment relief, income transfers, pensions, and basic health insurance. We use medical insurance coverage as a test case to evaluate whether it alleviates the financial impact of Sino–U.S. trade frictions. Based on whether household members are covered by medical insurance, we divide the sample into insured and uninsured households and re-estimate the model (Table 10).

**Table 10:** Heterogeneity by health insurance coverage.

	Households with medical insurance	Households without medical insurance
Treated*Post	0.0393^**^	0.4886^***^
	(0.0189)	(0.1776)
*Control Variables*	Yes	Yes
Time Effects	Yes	Yes
Individual Effects	Yes	Yes
*Coefficient*	1.4712^***^	0.6777
	(0.0989)	(0.4827)
*N*	20,440	897
*R* ^*2*^	0.0394	0.2902

Among uninsured households, the coefficient on the key interaction term is 0.4886 and significant at the 1% level, indicating a sharp increase in financial vulnerability. Among insured households, the coefficient remains positive and significant but is lower by about 0.45, suggesting that medical insurance mitigates the adverse impact of trade frictions on household finances.

The mechanism is intuitive. Trade frictions reduce household income and elevate unemployment risks, weakening resilience. When health shocks occur, uninsured households must fully bear medical expenses, depleting savings and increasing debt, thereby compounding risks and elevating financial fragility. In contrast, insured households can rely on partial coverage of medical costs, avoiding additional financial drain and breaking the chain of risk accumulation. Furthermore, predictable medical expenditures allow insured households to concentrate savings on buffering income shocks, thereby mitigating the adverse impact of trade frictions on household economic security.

## 7. Conclusions and policy implications

Using CFPS data from 2014–2022 and employing a DID framework with the 2018–2019 U.S. tariff hikes as a quasi-natural experiments, this study yields three principal conclusions.

First, trade frictions significantly exacerbate the financial vulnerability of Chinese households. This finding indicates that external shocks not only disrupt macroeconomic performance but also transmit through multiple channels to the household sector, amplifying financial risks at the micro level. Second, the impact of trade frictions on household financial vulnerability is markedly heterogeneous. Specifically, urban households, households located in highly open regions such as the Yangtze River Delta and Pearl River Delta, households headed by less-educated individuals, households lacking business or property income, and households with insufficient social security coverage are more severely affected. This underscores the buffering role of demographic characteristics, economic structures, and social protection systems in mitigating the consequences of external shocks. Third, Trade frictions weaken household financial resilience mainly through employment and income shocks, with price and savings channels playing secondary roles, while asset volatility and especially debt burdens exert the weakest effects. To mitigate these effects and enhance household economic resilience, we propose:

First, strengthen targeted support for vulnerable households. Given that different household types vary significantly in their likelihood of falling into financial vulnerability under trade shocks, it is essential to focus on targeted support for urban households, households in highly open regions, low-education households, and those lacking business or property income and social security coverage. Specifically, in regions with high dependence on foreign trade such as the Yangtze River Delta and the Pearl River Delta, governments should provide employment subsidies and retraining funds to enterprises to prevent large-scale unemployment.

Second, enhance households’ multi-channel risk resilience. Since trade frictions increase household financial vulnerability through employment, income, consumption, debt, savings, and asset-price volatility, policies should help strengthen households’ ability to absorb consumption shocks. In particular, measures that ease the burden of essential daily expenditures—especially for households in highly exposed regions—can mitigate the rise in financial pressure caused by external shocks.

## Supporting information

S1 File Appendix 1 and Appendix 2. (DOCX)
