# Supplementary material for: The impact of trade frictions on the financial vulnerability of Chinese households
Source: PLoS One. 2026 Apr 10;21(4):e0333713. doi: 10.1371/journal.pone.0333713 (PMC13068276; doi:10.1371/journal.pone.0333713)
Supplement: S1 File — (DOCX) [file pone.0333713.s001.docx]

Appendix 1*.* Descriptive statistics.

| Variable | Description | Mean | Standard deviation | Minimum | Maximum |
| --- | --- | --- | --- | --- | --- |
| *age* | Age of household head | 41.4669 | 12.6281 | 16 | 88 |
| *familysize* | Household size | 2.6767 | 1.3324 | 1 | 12 |
| *Uy* | Proportion of young children | 0.0173 | 0.0734 | 0 | 0.6667 |
| *Uo* | Proportion of elderly population | 0.1436 | 0.2627 | 0 | 1 |
| *edu* | Education level of household head | 0.2468 | 0.4312 | 0 | 1 |
| *marital* | Marital status of household head | 0.7928 | 0.4053 | 0 | 1 |
| *sex* | Gender of household head | 0.6552 | 0.4996 | 0 | 1 |
| *LEV* | Household financial vulnerability | 0.3750 | 0.4841 | 0 | 1 |
| *Post* | Time dummy variable | 0.3904 | 0.4879 | 0 | 1 |
| *Treated* | Treatment dummy variable | 0.3022 | 0.4592 | 0 | 1 |
| *urban* | Urban–rural status | 0.6582 | 0.4743 | 0 | 1 |
| *lny* | Log of household income | 11.1196 | 0.9618 | 0 | 16.2475 |

Appendix 2. Household income sources

|  | Labor income | Operating income | Property income |
| --- | --- | --- | --- |
| *with* | 89.50% | 32.43% | 19.51% |
| *without* | 10.50% | 67.57% | 80.49% |
